# Wettability Behaviour of Metal Surfaces after Sequential Nanosecond and Picosecond Laser Texturing

**DOI:** 10.3390/mi15091146

**Published:** 2024-09-12

**Authors:** Yin Tang, Zheng Fang, Yang Fei, Shuai Wang, Walter Perrie, Stuart Edwardson, Geoff Dearden

**Affiliations:** Laser Group, School of Engineering, University of Liverpool, Brownlow Street, Liverpool L69 3GQ, UK; y.tang49@liverpool.ac.uk (Y.T.); zheng.fang@liverpool.ac.uk (Z.F.); y.fei3@liverpool.ac.uk (Y.F.); s.wang162@liverpool.ac.uk (S.W.); wpfemto1@liverpool.ac.uk (W.P.); me0u5040@liverpool.ac.uk (S.E.)

**Keywords:** laser processing, surface modification, laser induced periodic surface structures (LIPSS), hydrophobic surface, post-processing, wettability

## Abstract

This study examines the wettability behaviour of 304 stainless steel (304SS) and Ti-6Al-4V (Ti64) surfaces after sequential nanosecond (ns) and picosecond (ps) laser texturing; in particular, how the multi-scale surface structures created influence the lifecycle of surface hydrophobicity. The effect of different post-process treatments is also examined. Surfaces were analysed using Scanning Electron Microscopy (SEM), a white light interferometer optical profiler, and Energy Dispersive X-ray (EDX) spectroscopy. Wettability was assessed through sessile drop contact angle (CA) measurements, conducted at regular intervals over periods of up to 12 months, while EDX scans monitored elemental chemical changes. The results show that sequential (ns + ps) laser processing produced multi-scale surface texture with laser-induced periodic surface structures (LIPSS). Compared to the ns laser case, the (ns + ps) laser processed surfaces transitioned more rapidly to a hydrophobic state and maintained this property for much longer, especially when the single post-process treatment was ultrasonic cleaning. Some interesting features in CA development over these extended timescales are revealed. For 304SS, hydrophobicity was reached in 1–2 days, with the CA then remaining in the range of 120 to 140° for up to 180 days; whereas the ns laser-processed surfaces took longer to reach hydrophobicity and only maintained the condition for up to 30 days. Similar results were found for the case of Ti64. The findings show that such multi-scale structured metal surfaces can offer relatively stable hydrophobic properties, the lifetime of which can be extended significantly through the appropriate selection of laser process parameters and post-process treatment. The addition of LIPSS appears to help extend the longevity of the hydrophobic property. In seeking to identify other factors influencing wettability, from our EDX results, we observed a significant and steady rate of increase in the carbon content at the surface over the study period.

## 1. Introduction

Inspired by surfaces in nature, such as lotus leaves and dahlia flowers [1], unique wettability properties such as superhydrophobicity and self-cleaning have attracted wide attention due to their potential importance for practical applications [2]. The wettability of a surface can be characterized by the contact angle (CA), which is less than 90° for hydrophilic surfaces and greater than 90° for hydrophobic surfaces. Based on the wetting models of Young [3], Wenzel [4], and Cassie-Baxter [5], a large number of different surface processes for creating hydrophobic surfaces have been studied including moulding [6], nanoparticle coating [7], electrospinning [8], chemical vapor deposition [9], electrochemical techniques [10], etc. A review of laser surface texturing to produce superhydrophobic surfaces has recently been published [11]. From the perspectives of different laser parameters [12,13,14,15,16], scanning strategies [13,17], substrate materials [15], post-treatment processes [18,19], and storage in different atmospheres [20,21] and environments [22,23], various applications of laser-textured hydrophobic surfaces and their performance in different scenarios have been explored such as anti-icing [24], anti-corrosion [25], and self-cleaning applications [26]. However, a current barrier to realising industrial applications of such laser-textured surfaces is a limited understanding of their wettability behaviour and development over meaningful timescales [27]. Previous studies on the effect of laser parameters and post-treatment were limited to less than two months duration, thus lacking long-term observation [13]. Post-treatment of the surface was required before each wettability test, which is not suitable for an actual working environment [26,28].

In this study, nanosecond (ns) and picosecond (ps) laser pulses were applied to 304 stainless steel (304SS) and Ti-6Al-4V titanium alloy (Ti64) to create multiscale surface texture and laser-induced periodic surface structures (LIPSS) by laser ablation. We define the latter as low spatial frequency LIPSS (LSFL), by virtue of their spacing dimensions being the order of the wavelength of light. The choice of materials was made to target potential application interests in bioengineering and aerospace. The wettability behaviour during natural ageing was monitored and evaluated over an extended period and for different post-process treatments, while also performing chemical element tracking by EDX. The primary aim was to understand the various stages of wettability behaviour over this lifecycle, while also considering aspects of performance including functionality, longevity, production efficiency, and some of the chemical changes taking place. Rather than seeking conditions for the highest CA (superhydrophobic), the study explored parameters and post-process treatments that could achieve hydrophobicity over an extended lifetime.

## 2. Materials and Methods

### 2.1. Materials and Preparation

Samples of dimensions 12 mm × 12 mm were prepared from flat sheet 304SS (Merseyside Metal, Wirral, UK) and grade 5 Ti64 (Alcoa, Manchester, UK). All samples were polished, ultrasonically cleaned in ethanol for 30 min, rinsed in deionized water, and then dried in ambient air before laser processing. The measured roughness of the polished samples was in the range $S_{a}$ = 0.14 ± 0.03 µm.

### 2.2. Pulsed Laser Surface Processing

**Figure 1:**
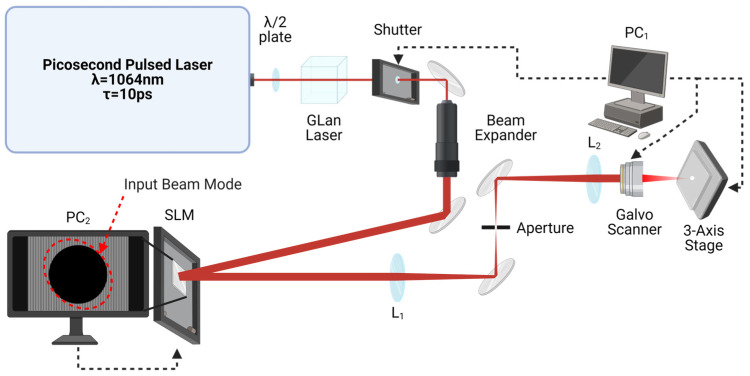
Schematic of optical setup used for ps laser processing (Created with BioRender.com). The beam was attenuated by a λ/2 plate and a Glan-Laser Calcite Polarizer transmitting horizontal polarization. The beam passes through a diffraction-limited beam expander (Rodenstock; M = x3) and is then modulated by a reflective phase-only SLM and input to a galvo scanner after passing through a 4f optical system. An aperture allows the shaped zero-order light to pass through for laser processing.

Our experiments used two different laser systems to structure the surfaces of 304SS and Ti64 in ambient air. Firstly, a ns-pulse fibre laser (RedENERGY G4 20W HS-L; λ = 1064 nm and variable pulse length τ = 9–200 ns, SPI Lasers TRUMPF, Southampton, UK) was used to ablate a double periodic surface structure in a grid pattern. The laser beam was guided and controlled by a galvanometer scanning head (XLR8-10, Nutfield, Hudson, NH, USA) with an integrated field correction lens (f = 160 mm). Samples were positioned at the focal plane by a Thorlabs LTS 150 XY stage and Thorlabs L490MZ motorized lab jack (Thorlabs, Ely, UK) under software control (WaveRunner, Version 3.5.5). For the case of a laser beam focal spot diameter at the sample surface of φₙₛ = 50 ± 2 µm, this would give a fluence of 5.76 Jcm^−2^. Secondly, a ps-pulse laser was used to create nanometre-scale LIPSS overlayed on the ns laser ablated structures. The beam from the ps laser (IC-355-800 ps; λ = 1064 nm; pulse length 10 ps; repetition rate 5 kHz, High Q Laser, Rankweil, Austria) had a quasi-Gaussian spatial mode, an output diameter of 2.3 mm, and linear horizontal polarization. As the beam mode was slightly elliptical, a spatial light modulator (X10468-03, 800 × 600 pixels, Hamamatsu Photonics, Hamamatsu, Japan) was applied to improve the roundness of the beam mode (transverse intensity profile) by addressing with a suitable hybrid Computer Generated Hologram (CGH) [29]. The sample surface was translated to the focal plane by a 3-axis (x, y, z) motion control system (ATS150 & AVS113, Aerotech, Pittsburgh, PA, USA). Using a 100 mm f-theta lens, the focal spot diameter was φₚₛ = 23.0 ± 0.5 µm. Figure 1 shows a schematic of the optical layout for ps laser surface LIPSS inscription. A detailed parametric study, ablation threshold tests, and evaluation of data from the literature yielded an optimized set of process parameters being selected for the two laser types and materials used in this study, as shown in Table 1.

### 2.3. Post-Process Treatment of Samples

Groups of the ns laser-processed samples were subjected to two different post-processing treatments: 1. low-temperature annealing (24 h at 200 °C, followed by natural cooling in ambient air) and 2. ultrasonic cleaning in an ethanol and water mixture (ratio 75:25) for 30 min at 40 °C followed by drying in ambient air. All post-processing treatments were conducted only once after laser texturing. A third (‘control’) group of samples had neither of the above post-process treatments applied.

### 2.4. Surface Characterization

**Figure 2:**
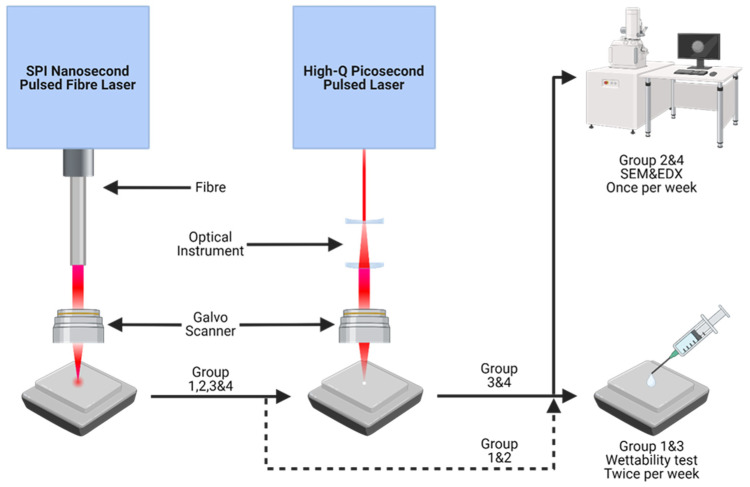
Schematic of the experimental process: laser process and surface property analysis methods (Created with BioRender.com). Four groups (1–4) of polished samples were processed with the ns laser to form the underlying micron-scale doubly periodic structure, while groups 3 and 4 were then also exposed to ps laser processing for LIPSS overlaying. For comparison, groups 1 and 3 were subjected to wettability tests twice a week, while groups 2 and 4 were measured for elemental concentration by EDX once a week. All samples were stored in ambient air during ageing.

The surface topography was evaluated using a white light interferometer optical profiler (WYKO NT1100, Veeco, Plainview, NY, USA) and a scanning electron microscope (JSM 6610, JEOL, Tokyo, Japan). The wettability of the surface was analysed by capturing the static contact angle θ image of 10 µL deionized water droplets with a drop shape analyser system (FM40 EasyDrop, Kruss, Hamburg, Germany) and a USB CMOS camera (DCC1545M, Thorlabs, Lübeck, Germany) while measuring the droplet contact angle with Image analysis software (ImageJ, Version 1.48). All samples were dried under a hot air gun at 150 °C for 5 min after wettability measurements. The experimental process diagram is shown in Figure 2. Chemical composition analysis of the sample surface was conducted using energy dispersive X-ray (EDX) spectroscopy (Xplore EDS detector, Oxford Instruments, High Wycombe, UK) with AZtec software (Version 6.1). The analysis parameters of work distance (10 mm), beam voltage (20 kV), EDS resolution (1024 pixels), acquisition time (288.4 s), channel number (1024), process time (6), and pixel dwell time (50 µs) were standardised during each EDS scanning. Wettability measurements were conducted on all samples twice a week, and separate groups were located for single weekly SEM and EDX data acquisitions.

## 3. Results

### 3.1. Surface Topography

**Figure 3:**
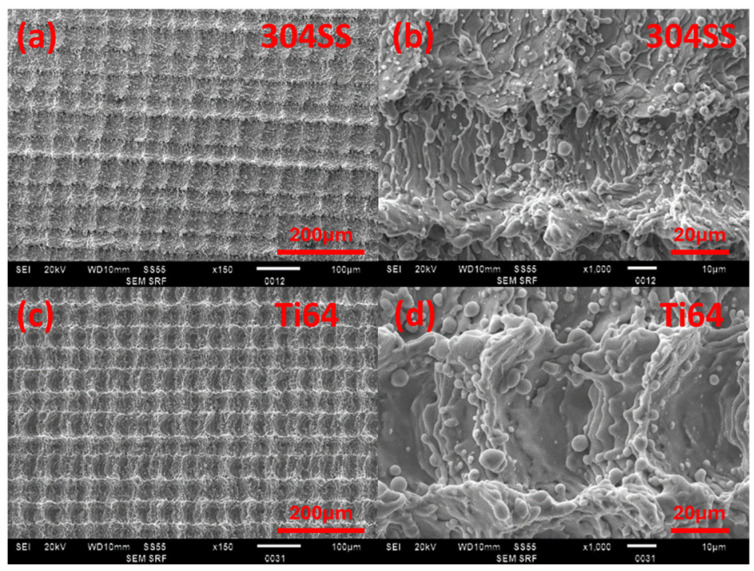
SEM images of ns laser processed 304SS and Ti64 surfaces. (**a**,**c**) are ×150 magnification, while (**b**,**d**) are ×1000 magnification.

The average surface roughness of grid structures created by ns laser ablation was measured to be R_a_ = 3.4 ± 0.6 µm and R_q_ = 3.5 ± 0.8 µm for 304SS and R_a_ = 7.5 ± 0.8 µm and R_q_ = 7.8 ± 1.2 µm for Ti64. Figure 3a,c shows the low-magnification SEM images of 304SS and Ti64, respectively. The higher magnification images in (b) and (d) show evidence of melting, which is to be expected when processing with longer ns pulse lengths.

**Figure 4:**
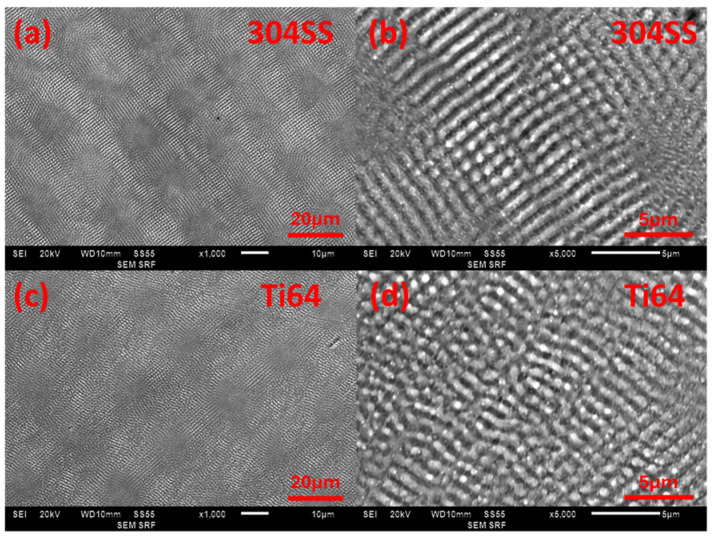
SEM image of LIPSS on 304SS and Ti64 surfaces: (**a**,**c**) ×1000 magnification and (**b**,**d**) ×5000 magnification.

The ability to create clear LIPSS formation with ps laser exposure was first tested on polished samples. Figure 4a–d displays SEM images of LIPSS on 304SS and Ti64. To maintain the coupling strength for LIPSS formation during scanning, the sample was rotated by 90° to produce orthogonal LIPSS. The higher magnification SEM images (b) and (d) demonstrate LIPSS with good finesse. From Wyko surface analysis, the average height of LIPSS on 304SS is h = 30 ± 10 nm and on Ti64, h = 50 ± 10 nm.

**Figure 5:**
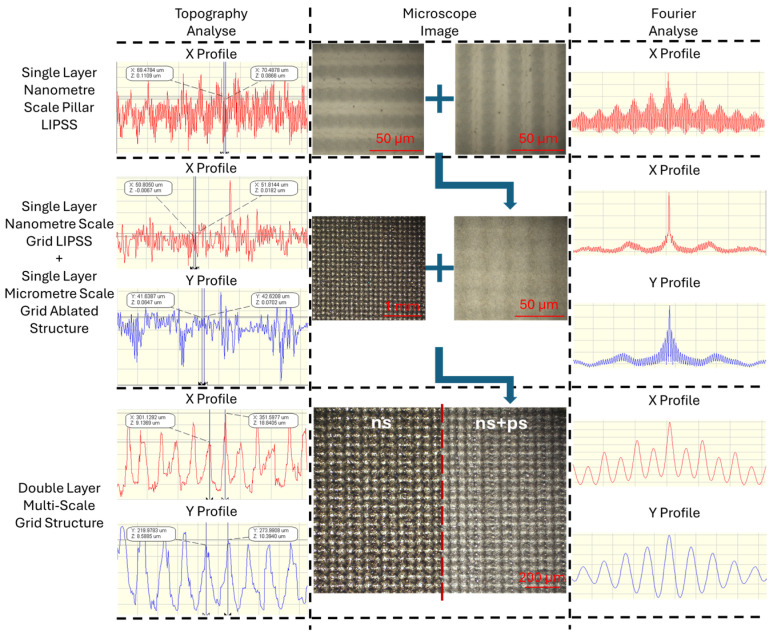
Surface topography 2D analyses, microscope images, and topography Fourier transform analyses of laser-textured functional surfaces before and after adding ps laser-generated LIPSS.

Figure 5 displays the observed topography curves of the single-scale structures and multi-scale structures, alongside their microscope images and Fourier transform analysis of the topography. The data confirm that the LIPSS created using the ps laser belong to the category of LSFL, as their pitch Λ = 1.05 ± 0.10 µm is close to the laser wavelength. The size of the crosshatch structures is close to the hatch distance. Fourier transform analysis shows that the addition of LIPSS does not significantly affect the average surface roughness, yet microscope images show that the overlay of LIPSS impacts the characterisation of the samples by smoothing ablation profiles and reducing optical scatter.

### 3.2. Long-Term Wettability Behaviour of Ns Laser-Ablated 304SS Surfaces for Different Post-Process Treatments and Conditions

**Figure 6:**
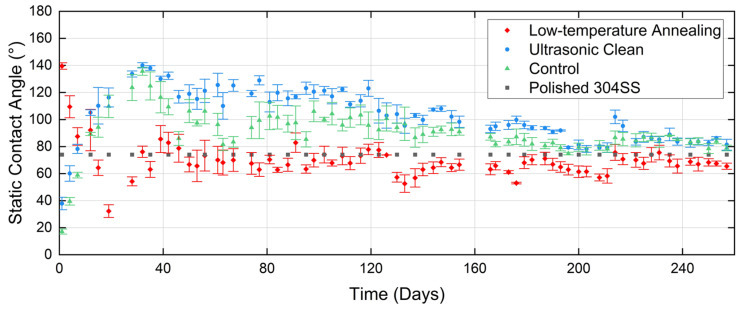
Long-term wettability behaviour of ns laser ablated 304SS surfaces, for different post-process treatments: CA measured during the period up to 257 days after exposure.

Figure 6 shows the variation with time of the measured CA of ns laser textured 304SS for each of the post-process treatments: annealing and ultrasonic cleaning. A control group with neither treatment applied is included for comparison, as is the static contact angle of the polished unprocessed batch measured at the beginning of the experiment (74.1°). Clear transitions from hydrophilic to hydrophobic (or vice versa) properties are observed.

From Figure 6, five key stages of CA development during this lifecycle can be discerned, primarily in relation to ultrasonically cleaned samples and the control group.

1. Wettability transition stage (end of post-process treatment to the 32nd day): During this period, the surface static CA steadily increases, transitioning from hydrophilic to hydrophobic and reaching a peak value of 140° by day 32 (~135° for the control group). In contrast, samples subjected to oven annealing are hydrophobic (CA = 140°) when measured after the treatment, but after which the CA reduces, transitioning to hydrophilic by day 7 and reaching 30° by day 19, then rising to around the CA of the polished (unprocessed) sample group by day 32;
2. Wettability ‘rebound’ stage (days 32–42): After reaching its peak, the CA exhibits a rebound effect (decrease in CA): the reduction is around 20° for samples treated with ultrasonic cleaning, compared to an average of 50° for the control group. Meanwhile, the CA for the oven-annealed group briefly dips below the polished sample level, before then rising above it to a peak value of 85°;
3. Hydrophobicity stabilization stage (days 42–119): This 77-day period represents the useful hydrophobic “service life” of the samples, during which the CA fluctuates within a certain range but remains hydrophobic throughout. The CA performance of samples treated with ultrasonic cleaning is superior to the other groups, with the CA remaining around 120° ± 10°, compared to the control group value of 100° ± 10°. The oven-annealed group shows the CA fluctuating in the range of 70° ± 10°;
4. Hydrophobicity decline stage (days 119–196): During this 77-day period, the CA gradually reduces to values close to 90° for the ultrasonically cleaned group, by which time that of the control group reduces to values less than the 90° transition;
5. Hydrophobicity termination stage (days 196–257): This stage marks the complete termination of hydrophobicity without reoccurrence. The CA stabilizes between 80° and 90°, though it is still slightly higher than the polished surface’s contact angle of 74°.

### 3.3. Wettability Time-Dependent Behaviour of Ns and Ns + Ps Laser Structured Surfaces

**Figure 7:**
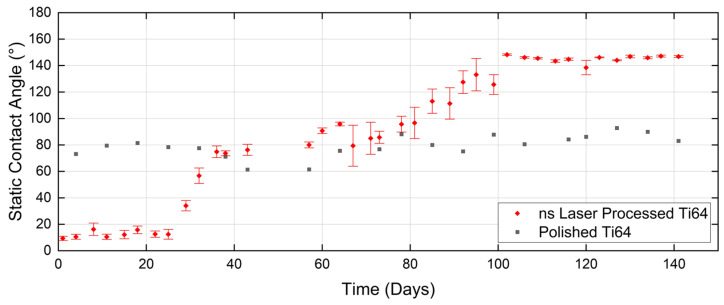
Wettability behaviour of ns laser processed Ti64 surfaces over a period of 141 days.

**Figure 8:**
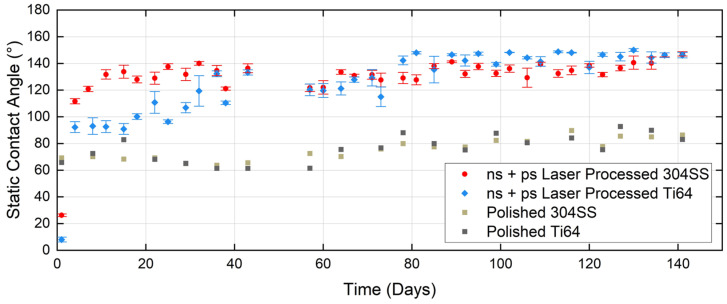
Wettability behaviour of ns + ps laser processed 304SS and Ti64 surfaces over a period of 141 days.

Since the experiment described in the previous section showed the positive effect of ultrasonic cleaning and superior CA performance of that sample group over the lifecycle, all subsequent samples were ultrasonically cleaned after laser processing. In this section, the long-term wettability behaviour of ns and ns + ps laser-processed surfaces of 304SS and Ti64 samples, following ultrasonic cleaning, is examined and compared. For ns laser-processed Ti64, as shown in Figure 7, a slow increase in CA occurred in the early stage. The hydrophobic transition began later, but the duration was significantly longer than that of 304SS (60 days). The CA stabilized at 140° after 120 days. As shown in Figure 8, the addition of LIPSS on 304SS results in a rapid transition to hydrophobicity within 5 days, reaching 130° after 15 days and staying nearly constant with time. The hydrophobic lifetime is as long as 4.5 months, which is twice that for the ns laser-processed samples in the previous section. Figure 8 also shows a similarly rapid rise in CA observed for Ti64, but increasing more slowly up to 130° at 40 days after exposure and stabilising at 140° at 80 days. The CA performance of ns + ps laser processed Ti64 samples is superior to those processed only by ns laser, but inferior to that of 304SS. The hydrophobic transition takes only 3 days, reaching a CA of 120° in 40 days, before fluctuating between 120° and 140° similar to the ns + ps 304SS case. In comparison, the time required for the ns + ps laser-treated Ti64 to reach the stable stage is one-third of that for the ns laser-treated sample, while its service life is two months longer than that of the ns laser-treated case.

### 3.4. Chemical Composition Time-Dependent Behaviour of Ns and Ns + Ps Laser Structured Surfaces

It is widely accepted that the CA of a metal surface depends on both its physical properties (topology and morphology) and chemical properties. For example, Keitzig et al. [15] found that changes in the wetting behaviour of metals over a 30-day period, following femtosecond laser micro-structuring, correlated with the amount of carbon on the surface. They deduced that the time dependency of the CA is the combined effect of surface morphology and surface chemistry. Observation of the chemical composition of surfaces is therefore considered a useful technique in wettability studies to elucidate possible factors behind CA variation. Since water is a polar molecule, hydrophobicity is often considered to be related to the presence of organic matter [20,21,22,23]. A detailed treatment of the influence of laser micro-structuring on surface chemistry is beyond the scope of this study. Here, however, we used EDX to explore if any variations in elemental composition observed during the ageing of the laser-processed samples correlated with changes in CA.

**Figure 9:**
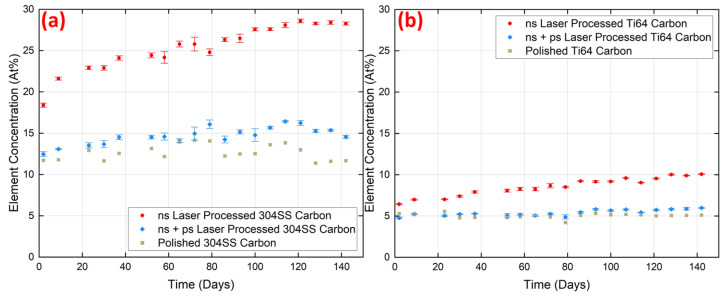
Comparison of time-dependent variation in carbon composition at the surface of samples processed by ns and (ns + ps) lasers, followed by ultrasonic cleaning: (**a**) 304SS and (**b**) Ti64.

Figure 9a,b shows the variation in carbon concentration (At%) at the surface of each material, measured over a 140-day period following each type of laser processing. Here, we were interested in tracking changes in the concentration with time, though it is also evident that in the ns laser process case, there is more carbon at the surface than immediately after dual ns + ps laser processing. For the ns laser process case, both 304SS and Ti64 surfaces exhibit significant changes in carbon content during the ageing process, and with similar trends. No other element underwent such a significant change over the period. For 304SS modified by ns laser only, the carbon concentration increased by 10 At% over 140 days of ageing, compared to 4 At% for the ns + ps laser modified surface (i.e., 2.5 times more). For the Ti64 modified by ns laser, the carbon content increased by 3.5 At% over the same period (3.5 times the 1 At% for the ns + ps laser case). The ns + ps laser-treated surface started with a lower carbon concentration and exhibited a relatively lower growth rate.

**Figure 10:**
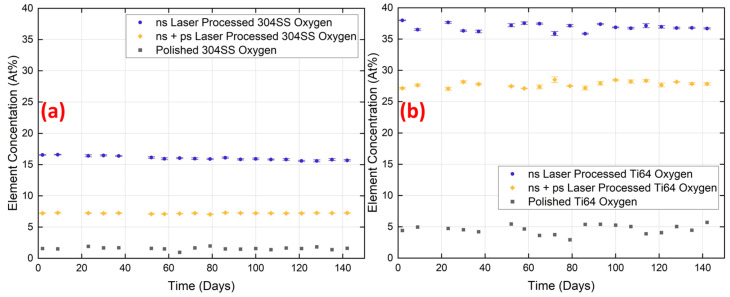
Comparison of time-dependent variation in oxygen composition at the surface of samples processed by ns and (ns + ps) lasers, followed by ultrasonic cleaning: (**a**) 304SS and (**b**) Ti64.

**Figure 11:**
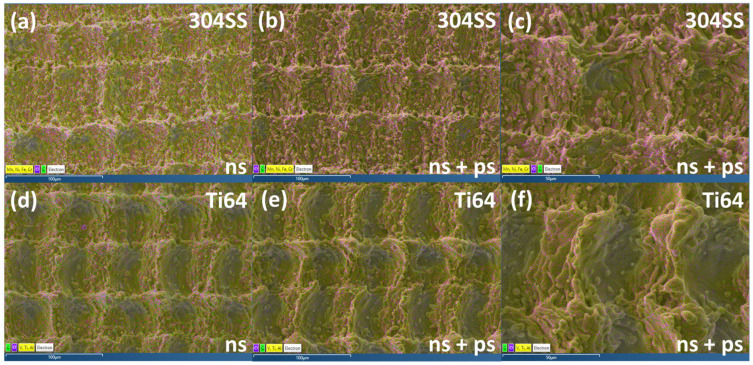
EDX element-layered distribution map on ns (**a**) 304SS and (**d**) Ti64 and ns + ps (**b**) 304SS and (**e**) Ti64 laser processed surface structure ×500 magnification and (**c**) 304SS and (**f**) Ti64 ×1000 magnification Ns laser processed structure has obvious wider and stronger carbon absorption and oxidises around the peak of the structure compared to the ns + ps laser processed structure.

Figure 10 shows the variation in oxygen concentration on the surfaces of the two materials during the 140-day ageing experiment. The ns laser structured materials exhibit only a minor reduction in oxygen content of less than 1 At%, whereas the content remains fairly constant for those structured by ns + ps laser, suggesting that the latter effectively reduces the thickness of the surface oxide layer. The fluctuation in oxygen content during the ageing period is negligible compared to carbon. Figure 11 shows the EDX results of the ns laser processed structure at ×500 magnification and that of the ns + ps laser processed structure at ×500 magnification and ×1000 magnification. Yellow represents the elements of the base material, purple represents the oxygen, and green represents the carbon. The oxygen and carbon are mainly concentrated on the peak of the structure. Also, the ns laser-processed surface has a larger area of element change than the ns + ps laser-processed structure and tends to expand to the surroundings of the peak. The orientation of the detector relative to the sample surface can affect the elemental imaging and measured elemental concentrations. This was checked by rotating samples through 180° to the detector, but the differences were found to be minimal, altering C and O concentrations by only ±1%, and still concentrated around the peak region of the structure.

**Figure 12:**
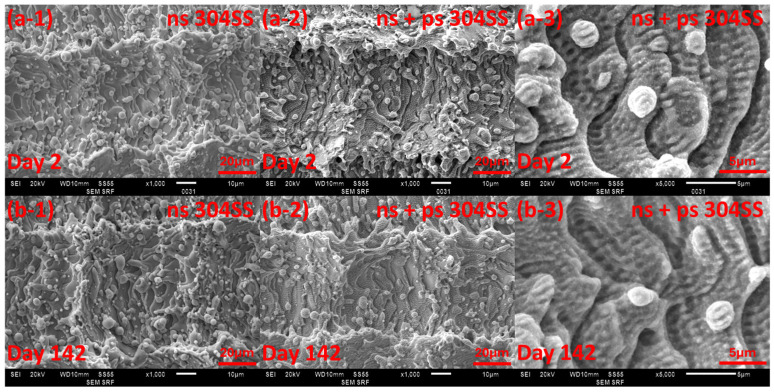
SEM images of 304SS on (**a**) day 2 and (**b**) day 142. ns (**a-1**,**b-1**) and ns + ps (**a-2**,**b-2**) laser processed surface structure ×1000 magnification and (**a-3**,**b-3**) ×5000 magnification.

**Figure 13:**
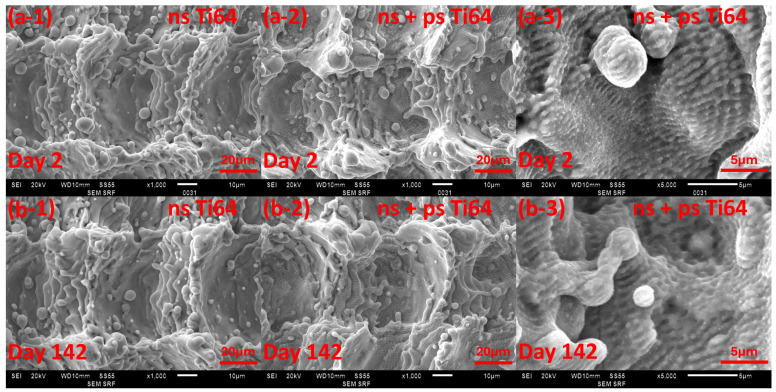
SEM images of Ti64 on (**a**) day 2 and (**b**) day 142. ns (**a-1**,**b-1**) and ns + ps (**a-2**,**b-2**) laser-processed surface structure ×1000 magnification and (**a-3**,**b-3**) ×5000 magnification.

We also considered the changes in surface topology during the experiment. The SEM images of the 304SS and Ti64 surface structures on day 2 and on day 142 are shown in Figure 12 and Figure 13 for comparison. In these, the structure did not appear to undergo any obvious changes. The surface roughness of the ns and ns + ps laser processed 304SS were measured to be R_a_ = 3.4 ± 0.6 µm and 3.2 ± 0.5 µm in day 2 and R_a_ = 3.2 ± 0.4 µm and 2.8 ± 0.4 µm in day 142, respectively. The ns and ns + ps laser-processed Ti64 were measured to be R_a_ = 7.5 ± 0.8 µm and 7.6 ± 0.8 µm on day 2 and R_a_ = 6.5 ± 0.4 µm and 6.6 ± 0.6 µm on day 142, respectively. Therefore, the roughness values measured on day 2 and day 142 were effectively equal within experimental error.

## 4. Discussion

Laser texturing on metals for improving hydrophobicity has shown conflicting results, even under similar experimental conditions [18]. This makes long-term observation of the surface wettability important, as it can help to elucidate the long-term effects of different treatments on the surface in actual applications. The functional tracking of this current study over many months confirms that the hydrophobicity eventually returns to a level close to that of a polished surface, indicating the potential applicable lifetime of the hydrophobic condition. In addition, another purpose of long-term wettability behaviour tracking is to understand ‘modes of failure’ in the hydrophobic condition and to provide guidance on failure management strategies. Although the time to failure varies with different materials, processing parameters, and application scenarios, such a gradual failure may not cause unpredictable loss of function and may give users a certain time margin to either replace a structured part before the property failure or to repeat the laser process.

Our results indicate that applying the combination of ns laser texture and ps laser generated LIPSS on top leads to a measurable improvement in hydrophobic performance. The use of ultrasonic cleaning treatment can improve the capacity to an extent. Given that the best hydrophobic performance comes from using 2 laser process steps and 1 additional treatment, it cannot be argued that the overall production efficiency has been improved, yet the quality of the hydrophobic surface has been enhanced, with its lifetime extended. The above combination of laser process and treatment steps also significantly shortens the overall processing time to reach a stable hydrophobic CA. Furthermore, since in Figure 8 the surface remains hydrophobic at the end of the experiment, we can only state that the hydrophobic lifetime of the ns + ps laser processed 304SS is at least twice that of the ns laser processed case. The fact that these benefits are reasonably consistent for both 304SS and Ti64 substrates suggests that the addition of LIPSS (with finer structure) is the underlying cause of the enhanced hydrophobicity. Our understanding of this is that the addition of LIPSS modifies the liquid-to-solid contact area in the Cassie-Baxter state. The ns + ps laser structure also appears more resistant to carbon contamination than the ns laser case. The reason for this may be related to physical adsorption. The adsorption kinetics are expected to be different for the cases of a polished unprocessed surface, a ns laser textured surface, and a ns + ps laser processed surface. The difference in carbon element growth may be due to the different adsorption models of the 2 laser-treated and 1 untreated surfaces.

Variations in surface roughness within the five-month duration of the experiment are effectively invariant within experimental error. This suggests that the variations in CA are primarily related to surface carbon content.

## 5. Conclusions

Both ns- and ps-pulsed lasers were employed to create multi-scale surface structures on 304SS and Ti64 by ablation. The surfaces were then subjected to different post-process treatments (only once), before being monitored long-term for CA and chemical content changes. The lifecycle wettability tests on ns laser-textured 304SS surfaces revealed five key stages of hydrophobic behaviour. The two post-process treatments each had a distinct influence on the CA variation. Ultrasonic cleaning slightly increased the hydrophobic CA values over the test cycle compared to the untreated control group, also resulting in a longer hydrophobic lifetime and superior CA stability. In contrast, the low-temperature annealing immediately results in strong hydrophobicity, which then transitioned in a matter of days to a hydrophilic state. Dual ns + ps laser-structured surfaces with LIPSS transitioned more rapidly to hydrophobic CA’s and led to far longer hydrophobic lifetime compared to samples only textured with the ns laser. For both materials treated by ns laser texturing, EDX analysis showed a significant (and steady rate of increase) in carbon content over the test cycle. Whereas, for the case of ns + ps laser processing, the surface structures showed much resistance to carbon adsorption, suggesting enhanced chemical stability. This study confirms that sequential (ns + ps) laser processing can significantly improve the hydrophobic lifetime of both metals used in the study, outperforming the ns laser-treated cases.

## Figures and Tables

**Table 1:** Laser processing parameters are applied for each material and laser type.

Material (Laser Type)	Laser Fluence $\left(J/{cm}^{2}\right)$	Scan Speed (mm/s)	Rep Rate and Pulse Duration (kHz; ns/ps)	Hatch Distance (μm)
304SS (ns)	12.1 ± 0.5	325	65; 65	50
Ti64 (ns)	7.1 ± 0.1	325	65; 65	50
304SS (ps)	1.20 ± 0.06	41.2	5; 10	20.6
Ti64 (ps)	0.82 ± 0.06	55	5; 10	25

## Data Availability

The original contributions presented in the study are included in the article, further inquiries can be directed to the corresponding author.
